# Functional solubilization of the β_2_-adrenoceptor using diisobutylene maleic acid

**DOI:** 10.1016/j.isci.2021.103362

**Published:** 2021-10-28

**Authors:** Clare.R. Harwood, David A. Sykes, Bradley L. Hoare, Franziska M. Heydenreich, Romez Uddin, David R. Poyner, Stephen J. Briddon, D.B. Veprintsev

**Affiliations:** 1 Centre of Membrane Proteins and Receptors (COMPARE), University of Birmingham and University of Nottingham, Midlands NG7 2UH, UK; 2 Division of Physiology, Pharmacology and Neuroscience, School of Life Sciences, Queen's Medical Centre, University of Nottingham, Nottingham NG7 2UH, UK; 3 Laboratory of Biomolecular Research, Paul Scherrer Institute, PSI, 5232 Villigen, Switzerland; 4 Department of Biology, ETH Zürich, 8093 Zürich, Switzerland; 5 School of Life and Health Sciences, Aston University, Birmingham B47ET, UK

**Keywords:** Biophysical chemistry, Membranes, Protein

## Abstract

The β2-adrenoceptor (β2AR) is a well-established target in asthma and a prototypical G protein-coupled receptor for biophysical studies. Solubilization of membrane proteins has classically involved the use of detergents. However, the detergent environment differs from the native membrane environment and often destabilizes membrane proteins. Use of amphiphilic copolymers is a promising strategy to solubilize membrane proteins within their native lipid environment in the complete absence of detergents. Here we show the isolation of the β_2_AR in the polymer diisobutylene maleic acid (DIBMA). We demonstrate that β_2_AR remains functional in the DIBMA lipid particle and shows improved thermal stability compared with the n-dodecyl-β-D-maltopyranoside detergent-solubilized β_2_AR. This unique method of extracting β_2_AR offers significant advantages over previous methods routinely employed such as the introduction of thermostabilizing mutations and the use of detergents, particularly for functional biophysical studies.

## Highlights

• DIBMA can be used to extract the human β_2_AR from mammalian cells
• DIBMALP-β_2_AR retains ligand binding ability and shows improved stability
• TR-FRET-based ligand binding methods avoid purification for DIBMALP-β2AR characterization

## Graphical abstract

**Figure:**
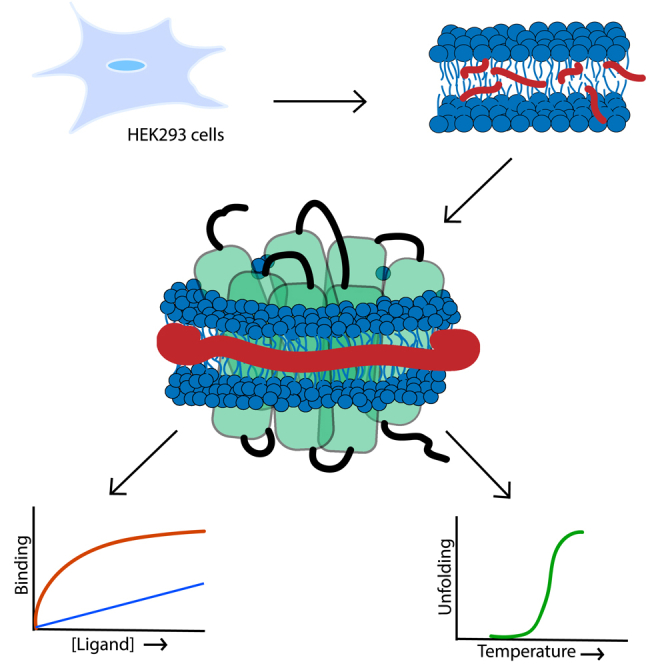

## Introduction

G protein-coupled receptors (GPCRs) are the largest family of membrane proteins within the human genome and are responsible for modulating a broad range of hormonal, neurological, and immune responses. It is well established that GPCRs have a large therapeutic potential. Indeed, GPCRs currently represent 34% of all US food and drug administration-approved drugs, with 475 drugs targeting over 100 diverse receptors (Hauser et al., 2017). The β_2_-adrenoceptor (β_2_AR) is a rhodopsin-like family GPCR (Schioth and Fredriksson 2005) and member of the adrenoceptor family, which signals primarily through coupling the heterotrimeric G_s_ protein. It is a well-established target for asthma and has become one of the most studied GPCRs with several structural (Wacker et al., 2010; Rasmussen et al., 2011; Bang and Choi 2015) and detailed biophysical studies (Manglik et al., 2015; Gregorio et al., 2017) into its activation mechanism.

A prerequisite for completion of biophysical and structural studies is the extraction and isolation of the β_2_AR from its cellular environment. Classically, this has involved the use of detergents; in the case of the β_2_AR and other GPCRs, *n*-dodecyl-β-D-maltopyranoside (DDM) is most often used (Munk et al., 2019). However, it is well established that detergent micelles do not recapitulate the environment of the cell membrane and, as such, protein stability is compromised. Moreover, there is strong evidence that phospholipid composition affects β_2_AR function (Dawaliby et al., 2016). Cholesterol in particular appears associated with the β_2_AR in crystal structures (Cherezov et al., 2007) and improves β_2_AR stability (Zocher et al., 2012) and function (Paila et al., 2011). Multiple studies (Leitz et al., 2006; Whorton et al., 2007) have mimicked the native membrane environment and improved protein stability through reconstitution of membrane proteins in liposomes, amphipols, or synthetic nanodiscs; however, these all require initial use of detergents to extract the membrane protein from the membrane.

Recently, it was discovered that styrene maleic acid (SMA) copolymer directly incorporates into biological membranes and self-assembles into native nanoparticles, known as Styrene Maleic Acid Lipid Particles (SMALPs) (Knowles et al., 2009) (Stroud et al., 2018), avoiding the use of detergents at all stages. This has provided a novel method for the solubilization of membrane proteins with their native receptor-associated phospholipids, although some preferential extraction of native lipids occurs (Barniol-Xicota and Verhelst 2021).

SMA has been used to solubilize a range of membrane proteins (Dorr et al., 2014; Gulati et al., 2014; Sun et al., 2018) including GPCRs (Jamshad et al., 2015; Bada Juarez et al., 2020) for both structural and biophysical studies. Such studies either improved protein stability compared with detergent or have allowed extraction of membrane proteins that were previously unstable in detergents. There is, however, evidence that the conformational flexibility of GPCRs within SMALPs is restricted (Mosslehy et al., 2019; Routledge et al., 2020), therefore differing from the native state of the protein. Furthermore, the high absorbance of SMA copolymer in the far-UV region makes optical spectroscopic studies of membrane proteins that are encapsulated within SMALPs challenging (Gulamhussein et al., 2019).

An alternative to SMA is diisobutylene maleic acid (DIBMA), a copolymer that was developed specifically for the extraction of membrane proteins from the intact bilayer (Oluwole et al., 2017). Compared with SMALPs, DIBMALPs are believed to have only a mild effect on lipid packing, be compatible with optical spectroscopy in the far UV range, and tolerate low millimolar concentrations of divalent cations (Oluwole et al., 2017). This makes DIBMALPs far more amenable for functional biophysical studies. DIBMALPs have been shown to contain lipids of the cell membranes using lipidomic approaches (Barniol-Xicota and Verhelst 2021). Despite the natural polydispersity in length of polymer molecules, DIBMALPs form a monodisperse in size population (Oluwole et al., 2017; Gulamhussein et al., 2019; Gulamhussein et al., 2020). Inclusion of integral membrane proteins such as OmpLA and α-synuclein did not affect their size distribution (Oluwole et al., 2017; Adão et al., 2020).

In this study we demonstrate isolation of the functional β_2_AR from the mammalian cell membrane using DIBMA, with improved thermal stability compared with conventional detergent-based methods.

## Results

### Extraction of the β_2_AR from membranes using DIBMA

**Figure 1:**
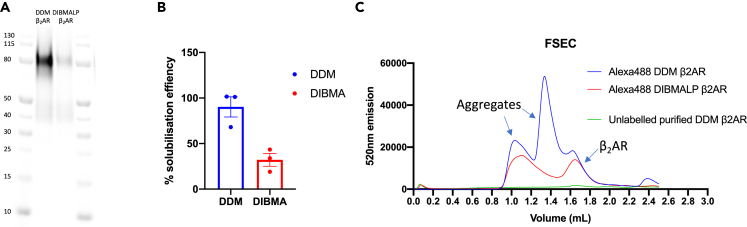
Solubilization of β_2_AR from the mammalian cell membrane using DDM and DIBMA (A) Representative in gel fluorescence (cy5) of purified DDM-β_2_AR and DIBMALP-β_2_AR. (B) Solubilization of DDM versus DIBMA, Alexa 488-labeled β_2_AR was quantified and normalised to the membrane content of β2AR (n = 3 ± SEM). (C) FSEC analysis of DDM-β_2_AR and DIBMA-β_2_AR samples using Yarra X300 column (mean of n = 3).

The β_2_AR was extracted from the membrane of mammalian (T-Rex-293) cells using either 1% DDM or 3% DIBMA (Figure 1A). Figure 1B shows a comparison of the solubilization efficiency of 1% DDM and 3% DIBMA as 90 ± 11% and 32 ± 7%, respectively. Figure 1C shows fluorescence size exclusion chromatography (FSEC) of these β_2_ARs. Figure 1C shows a peak at 1.6–1.8 mL, roughly 75 kDa, which corresponds to DDM-β_2_AR or DIBMALP-β_2_AR. In addition, there was a higher-molecular-weight peak for the DIBMALP-β_2_AR and two higher-molecular-weight peaks for DDM-β_2_AR. These peaks are presumed to correspond to protein aggregates. Peaks were confirmed by in-gel fluorescence (Figures S2A and S2B).

### DIBMALP-β_2_AR retains its pharmacology

A time-resolved fluorescence resonance energy transfer (TR-FRET)-based ligand binding assay was established to investigate if the β_2_AR remained functional when extracted from the HEK cell membranes into DIBMALPs. Förster resonance energy transfer (FRET) is the non-radiative transfer of energy from an excited donor fluorophore to a ground state acceptor fluorophore. Energy transfer will only occur when the fluorescent emission spectrum of the donor overlaps with the excitation spectrum of the acceptor fluorophore and these fluorophores are within ∼10 nm of each other. In this study, the SNAP tag on the N terminus of the β_2_AR was labeled with donor fluorophore terbium cryptate (Lumi4-Tb). Excitation of terbium cryptate using a laser allowed proximity of the β_2_AR to acceptor fluorophores fluorescent propranolol and the BIODIPY F-L cysteine dye to be quantified for ligand binding and thermostability assays, respectively. The specific labeling of the SNAP tag meant that it was not necessary to purify the β_2_AR in these studies. TR-FRET is becoming an increasingly used technique for ligand binding studies (Emami-Nemini et al., 2013).

**Figure 2:**
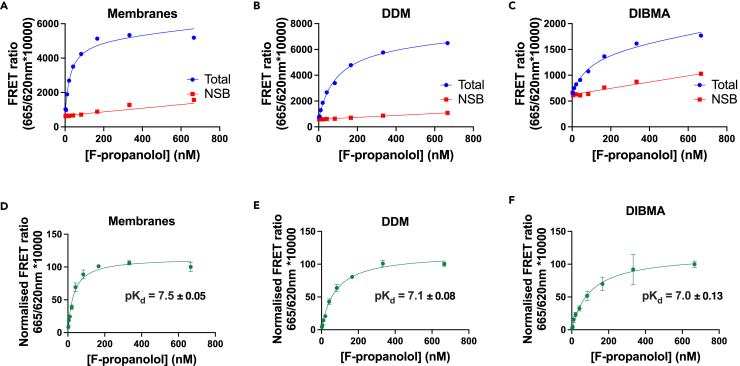
A comparison of F-propranolol binding to β_2_AR in membranes, DDM, and DIBMALPs (A–C) Representative F-propranolol (2–666 nM) equilibrium saturation plots showing total and non-specific binding to the β_2_AR in (A) HEK cell membranes, (B) DDM, and (C) DIBMALPs, n = 1. (D–F) Saturation binding curves showing specific binding and associated affinity (pK_d_) values for F-propranolol binding to the β_2_AR in (D) HEK cell membranes, (E) DDM, and (F) DIBMALPs; curves show combined normalized data mean ± SEM, n = 3.

Figure 2 shows saturation binding experiments for the fluorescent antagonist *S*-propranolol-red-630/650 (F-propranolol) binding membrane-β_2_AR, DDM-β_2_AR, and DIBMALP-β_2_AR. The β_2_AR retained ligand binding ability when extracted from the membrane using both DDM and using the copolymer DIBMA. These data showed comparable affinities for F-propranolol binding to the β_2_AR in membranes (pK_d_ = 7.50 ± 0.05), DDM (pK_d_ = 7.10 ± 0.08), and DIBMA pK_d_ = 7.00 ± 0.13), although with slightly reduced affinity in DIBMA compared with membranes (P = 0.02, one-way ANOVA and Tukey's multiple comparison).

**Figure 3:**
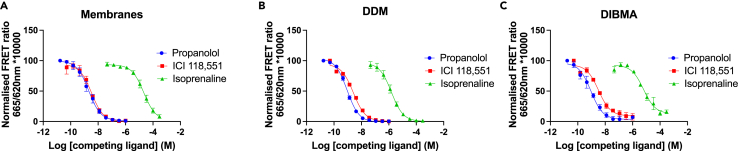
Competition TR-FRET ligand binding studies using F-propranolol as a tracer and unlabeled propranolol, ICI 118,551, and isoprenaline as competitors (A) β_2_AR membranes, (B) DDM-β_2_AR, (C) DIBMALP-β_2_AR curves show normalized combined data of n = 3, error bars show ±SEM.

In order to better understand if the conformational state of the receptor or its ability to adopt different states in DIBMALPs was affected we investigated its pharmacology using the full agonist isoprenaline, the antagonist propranolol, and the inverse agonist ICI 118,551 in equilibrium competition binding assays using F-propranolol as the tracer (Figure 3). Increasing concentrations of each competing ligand produced a reduction in the specific binding of F-propranolol to the β_2_AR in membranes, DDM, and DIBMALPs with largely comparable pK_i_ values (Table 1). The only statistically significant difference was between isoprenaline binding to the β_2_AR found in membranes versus the DDM-solubilized β_2_AR (p = 0.03) (one-way ANOVA and Tukey's post hoc). The slopes of all curves were similar to 1.

**Table 1:** Ligand binding parameters of different preparations of β_2_AR

	Membranes			DDM			DIBMA		
	pIC_50_	pK_i_	Slope	pIC_50_	pK_i_	Slope	pIC_50_	pK_i_	Slope
Propranolol	8.7 ± 0.13	9.5 ± 0.03	1.0 ± 0.02	9.0 ± 0.04	9.5 ± 0.03	1.2 ± 0.04	9.1 ± 0.10	9.6 ± 0.10	0.8 ± 0.30
ICI 118,551	8.5 ± 0.10	9.3 ± 0.15	1.1 ± 0.22	8.5 ± 0.02	8.9 ± 0.10	1.0 ± 0.06	8.3 ± 0.15	9.1 ± 0.06	1.3 ± 0.23
Isoprenaline	4.7 ± 0.12	5.5 ± 0.20	1.1 ± 0.11	5.8 ± 0.06	6.3 ± 0.13	1.1 ± 0.09	5.1 ± 0.18	5.8 ± 0.10	1.1 ± 0.15

Values shown are mean of n = 3 individually fitted curves ±SEM, as determined by TR-FRET competition binding assays.

### DIBMALP-β_2_AR shows improved stability

**Figure 4:**
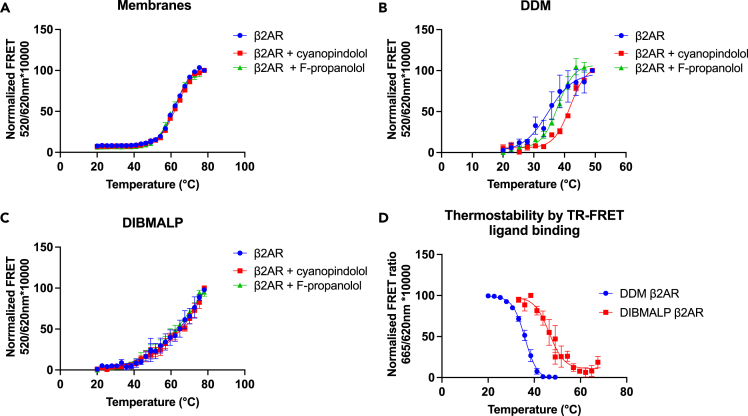
Thermostability of membrane, DDM and DIBMALP preparations of β2AR (A–C) ThermoFRET thermostability curves in (A) β_2_AR membranes, (B) DDM-solubilized β_2_AR, (C) DIBMALP-β_2_AR in the presence and absence of cyanopindolol (100 μM) and F-propranolol (200 nM). (D) β_2_AR and A_2_AR TR-FRET thermostability curves obtained by measuring reduction in fluorescent F-propranolol (200 nM) and F-XAC (200 nM) binding. All curves show normalized combined data, data points show mean ± SEM, for n = 3.

Next, we investigated the thermostability of the DIBMALP-β_2_AR using a novel ThermoFRET assay (Figure 4, Table 2). Labeling of the SNAP tag on the N terminus of the receptor with Lumi4-Tb allowed thermostability to be investigated without purifying the receptor. β_2_AR unfolding was initially measured by quantifying TR-FRET between Lumi4-Tb and BODIPY FL L-Cystine that covalently reacted with cysteines that become exposed as the receptor unfolded (Tippett et al., 2020).

**Table 2:** Thermal stability of different preparations of β_2_AR

	Tm (^°^C) ThermoFRET	Tm(^°^C) F-ligand
Membrane β_2_AR	62.4 ± 0.2	–
Membrane β_2_AR + F-propranolol	61.6 ± 0.4	60.1 ± 0.6^a^
Membrane β_2_AR + cyanopindolol	63.0 ± 0.4	–
DDM β_2_AR	35.2 ± 2.4	–
DDM β_2_AR + F-propranolol	37.8 ± 0.4	36.0 ± 0.6^a^
DDM β_2_AR + cyanopindolol	41.9 ± 0.1	–
DIBMALP β_2_AR	–	46.8 ± 2.1^a^
DIBMALP A_2A_ + F-XAC	–	44.8 ± 0.7^b^

Reported error values are **SEM**.

a β_2_AR thermostability was measured using F-propranolol dissociation.

b DIBMALP-A_2A_ thermostability was measured using F-XAC dissociation.

Figure 4B shows the Tm of DDM-solubilized β_2_AR as 35.2 ± 2.4°C. Ligand-induced shifts in thermostability were seen when the DDM-solubilized β_2_AR was incubated with F-propranolol (Tm = 37.8 ± 0.4°C, p > 0.05) and cyanopindolol (Tm = 41.9 ± 0.1°C, p = 0.04) (one-way ANOVA and Tukey's multiple comparison test). Figure 4A shows the Tm of β_2_AR in the membrane environment as 62.42 ± 0.2°C. No ligand-induced shift was observed when β_2_AR membranes were pre-incubated with F-propranolol or cyanopindolol; this suggests the unfolding of the receptor itself is not directly measurable and perhaps that these data show the disintegration of the membrane itself. Figure 4C shows TR-FRET thermostability data for the DIBMALP- β_2_AR; these data did not fit a Boltzmann sigmoidal curve as the top end of the temperature range did not plateau. No effect on any part of the curve was observed with the addition of F-propranolol or cyanopindolol. Therefore, as was the case in membranes, the observed thermostability changes in DIBMALPs likely reflect the melting of the lipid particles as opposed to the receptor itself.

We then investigated the thermostability of the β_2_AR by measuring the reduction in TR-FRET binding of F-propranolol over an increasing temperature range (Figure 4D). This gave the Tm values of 60.1 ± 0.6°C for membrane-β_2_AR (60.1 ± 0.6°C) and DDM-β_2_AR (36.0 ± 0.6°C) similar to those obtained using BODIPY FL L-Cystine in the presence of F-propranolol. Unpaired two-tailed t tests showed no statistically significant differences between membrane-β_2_AR + F-propranolol or DDM-β_2_AR + F-propranolol Tm values obtained using ThermoFRET versus that obtained by measuring the decrease in F-propranolol ligands binding.

Thermostability of DIBMALP-β_2_AR measured by the decrease in F-propranolol binding gave a curve that could be fitted to a Boltzmann with a Tm value of 46.8 ± 2.1°C. This Tm value is statistically significant from that of membrane-β_2_AR (p = 0.0002) and DDM-β_2_AR (p = 0.0009) obtained by the same method (one-way ANOVA and Tukey's multiple comparison test). Therefore, the DIBMALP-β_2_AR shows approximately 10°C improved stability over the conventional DDM-β_2_AR. We also observed differences in the slopes of DIBMALP-β_2_AR and DDM-β_2_AR thermostability curves obtained by this method; these were −3.2 and −2.7, respectively. In addition, we investigated the thermostability of another rhodopsin-like GPCR, the adenosine 2A receptor (A_2A_R), when solubilized into a DIBMALP using fluorescent adenosine receptor antagonist (F-XAC) (Hello Bio, UK). Measuring the reduction in F-XAC bound to A_2A_R over an increased temperature range gave a Tm value of 44.8°C ± 0.7, which was not statistically significantly different from that of the DIBMALP-β_2_AR.

## Discussion

The β_2_AR has become the prototypical GPCR for understanding GPCR structure and the molecular basis of signaling (Bang and Choi 2015; Gregorio et al., 2017); these studies have all required the use of detergents to extract the β_2_AR from the plasma membrane. Detergents do not recapitulate the complexity of the native membrane environment and are known to damage membrane proteins. Here, we demonstrate that the polymer DIBMA can be used to extract the β_2_AR from the plasma membrane, together with its native phospholipids, avoiding the use of detergents at any stage.

Similarly to Gulamhussein et al. (2020), we show that DIBMA can be used to extract GPCRs from cell membranes and that the solubilization efficiency of DIBMA is lower than that of SMA or in our case the detergent DDM. We then used TR-FRET ligand binding studies to show that the β_2_AR remained functional inside the DIBMALP (Figures 2 and 3). Ligand binding data showed comparable affinity (pK_d_/K_i_) values for the β_2_AR binding F-propranolol, propranolol, ICI 118,551, and isoprenaline solubilized in DIBMA compared with membranes. Although the difference in pK_d_ values for F-propranolol binding membranes-β_2_AR (7.5 ± 0.05) and DIBMALP-β_2_AR (7.0 ± 0.13) was statistically different (P = 0.02), this is only a 3-fold difference, and the pharmacological importance of this remains to be seen. There was no statistical difference between F-propranolol pK_d_ values for DDM-β_2_AR and DIBMALP-β_2_AR. All ligand binding curves showed one phase binding and a slope of 1 indicating no co-operativity of ligand binding.

Although the pK_d_ values for different preparations of the receptor were comparable, the signal amplitude obtained for F-propranolol binding DIBMALP-β_2_AR in TR-FRET experiments was 3-fold lower than for membranes-β_2_AR. This reduction in signal amplitude could be due to an effect of the DIBMA polymer on the TR-FRET, for example, fluorescence quenching. Alternatively, it could reflect that a lower fraction of the ligand binding capable β_2_AR receptors are present compared with the amount of Tb3+ labeled receptor molecules. However, it should be noted that the assay window for DDM-β_2_AR was higher than that of membranes, whereas it would be expected that less β_2_AR is functional, suggesting that the solubilization environment can influence the observed signal amplitude. Although the concentration of β_2_AR used in each experimental condition was quantified using 620 nm emission of Lumi4-Tb, it was not possible to account for difference in Lumi4-Tb quantum yield in the membrane, DDM and DIBMALP environments.

It has been shown that the conformational changes of another class A GPCR, Rhodopsin II, in response to activation by light are restricted in SMALPs (Mosslehy et al., 2019). We chose to study the binding of a full agonist (isoprenaline), antagonist (propranolol), and inverse agonist (ICI 118,551) to be able to ascertain if conformational states of the β_2_AR differed in a membrane, DDM micelle, or DIBMALP environment. A substantial increase or decrease in pK_i_ value would demonstrate an increase or decrease in the population of the receptors in the conformational state stabilized by the ligand, and therefore a difference in the conformational landscape of the receptor. As there was no statistically significant difference in pK_i_ values between membrane-β_2_AR and DIBMALP-β_2_AR, it can be concluded that the DIBMALP-β_2_AR represents the native conformational landscape of the β_2_AR. The difference in pK_i_ values between DDM-β_2_AR (6.3 ± 0.13) and membrane-β_2_AR (5.5 ± 0.2) for isoprenaline was statistically significant (p = 0.03); this may indicate a change in the conformational state of β_2_AR in the DDM micelle compared with its native conformational state. Propranolol, ICI 118,551, and isoprenaline pK_i_ values obtained in this study are in line with the previous studies that investigated the affinity of these compounds for the β_2_AR (Baker 2005; Sykes et al., 2014).

Furthermore, we employed a ThermoFRET-based thermostability assay to investigate the stability of the DIBMALP-β_2_AR compared with the DDM-β_2_AR. We show that the thermostability of DIBMALP-β_2_AR is 10°C higher than that of the DDM-β_2_AR. It was not possible to find any thermostability data for the β_2_AR in synthetic nanodiscs; however, the only other method to show a similar (11°C) increase in thermostability for β_2_AR is that of thermostabilizing mutations (Serrano-Vega and Tate, 2009). Since these mutations also lead to a shift in the β_2_AR's conformational landscape to the antagonist-bound and inactive form, the DIBMALP-β_2_AR offers a clear advantage for study of receptor function.

Moreover, thermostability data for DIBMALP-β_2_AR using F-propranolol showed a Tm value that was very similar to the Tm of DIBMALP-A_2A_R. In addition, no shift in thermostability was observed for DIBMALP-β_2_AR preincubated with F-propranolol or the high-affinity antagonist cyanopindolol. It therefore seems likely that this Tm value of ∼45°C for DIBMALP-β_2_AR corresponds to the melting temperature of DIBMALP. Interestingly, this Tm value of ∼45°C is lower than that of ∼50°C reported for SMALP-A_2A_R extracted from yeast membranes using a radioligand-based thermostability assay but slightly higher than ∼42°C A_2A_R extracted from mammalian (HEK293) membranes (Jamshad et al., 2015). The Tm of 60.2 ± 0.2°C seen for β_2_AR in membranes was also unaffected by the presence of F-propranolol and cyanopindolol. As this Tm of 60.2 ± 0.2°C is statistically significant from that of the DIBMALPs it seems likely that the Tm of ∼45°C corresponds to disruption of the protein-lipid-polymer particles, whereas the Tm of 60.2 ± 0.2°C corresponds to the melting or disruption of the membrane itself. We also noted a shallower slope for DIBMALP-β_2_AR (−3.2) compared with DDM-β_2_AR (−2.7); this broader transition may reflect the more heterogeneous nature of DIBMALPs compared with the detergent micelle.

In summary, here we show the utility of the copolymer DIBMA to solubilize the β_2_AR in a functional form. We show that this method offers improved stability over the use of the conventional detergent DDM and has allowed us to maintain the native environment and ligand binding activity of the β_2_AR. This could therefore provide an improved solubilization method for structural and biophysical studies. Moreover, we demonstrate this using novel TR-FRET ligand binding-based methods that should allow for easier screening of membrane protein solubilization conditions and anticipate that this approach could be applied to other GPCRs.

### Limitations of the study

As discussed above, one limitation of the study is that we cannot be certain about the cause of the decreased signal window in the ligand binding assays for DIBMALP-β_2_AR versus membrane-β_2_AR and DDM-β_2_AR. This could be indicative of less DIBMALP-β_2_AR being functional compared with in membranes, although there could be a number of other reasons such as decreased quantum yield of either donor or acceptor, as discussed. Furthermore, although we showed that DIBMALP-β_2_AR retains ligand binding, we have not tested other functionality of the receptor such as its ability to activate G proteins or recruit arrestins. Such studies would require the purification of G proteins, which was beyond the scope of this study.

## STAR★Methods

### Key resources table

**Table:** 

REAGENT or RESOURCE	SOURCE	IDENTIFIER
**Bacterial and virus strains**		

One Shot^TM^ TOP10 chemically competent E.coli	Invitrogen	C404010

**Chemicals, peptides, and recombinant proteins**		

SNAP-Lumi4-Tb	Cis bio	SSNPTBX
n-Dodecyl- β-D-maltopyranoside (DDM)	Anatrace, OH, US	D3010S
Diisobutylene Maleic acid (DIBMA)	Anatrace, OH, US	BMA1011
5% Magstrep ‘type3’ XT magnetic bead suspension	IBA Lifesciences, DE	2-4090-002
633/650 S-propranolol-red	CellAura, UK, supplied by Hello Bio, UK	Cat no. HB7817
Fluorescent XAC CA200634	CellAura, UK, supplied by Hello Bio, UK	Cat no. HB7814
ICI, 118 551 hydrochloride	Selleckchem, US	Cat no. S8114 1217094-53-5
Isoprenaline hydrochloride	Sigma	Cat no. I5627 CAS-51-30-9
(s)-(-)-Propranolol hydrochloride	Tocris, UK	Cat no. 0834 CAS 4199-10-4
Cyanopindolol hemifumerate	Tocris, UK	Cat no. 0993 CAS 69906-86-1
BODIPY^TM^ FL L- Cystine dye	Molecular Probes, US	B20340
NuPAGE^TM^ MOPS SDS running buffer (x20)	Invitrogen	NP0001

**Experimental models: Cell lines**		

T-Rex^TM^-293 cells (parent cell line)	Invitrogen	Cat.no. R71007
Stable cell line T-Rex^TM^-293 expressing TS-SNAP- β_2_AR	plasmid generated in this study	This study
Stable cell line T-Rex^TM^-293 expressing TS-SNAP- A_2A_	plasmid generated in this study	This study

**Oligonucleotides**		

CMV forward sequencing primer	Genewiz, UK	CGCAAATGGGCGGTAGGCGTG
BGH reverse sequencing primer	Genewiz, UK	TAGAAGGCACAGTCGAGG

**Recombinant DNA**		

TS-SNAP- β_2_AR in pcDNA4/TO	This study	This study, SI
TS-SNAP- A_2A_ in pcDNA4/TO	This study	This study, SI

**Software and algorithms**		

GraphPad Prism 8.0.0	GraphPad software, CA, US	www.graphpad.com
PHERAstar v5.41.	BMG, UK	BMG Pherastar FSX platereader

**Others**		

Shimadzu Prominence modular HPLC system	Shimadzu, Kyoto, Japan	Prominence modular HPLC system
Yarra 1.8μm SEC-x300 2.5mL column	Phenomenex, CA, US	00H-4743-E0-SS
TruPage^TM^ Precast Gels 4-20%.	Sigma	PCG2008
PHERAstar ® *FSX* equipped with Time Resolved Fluorescence lasers and module, and TR337/665/620 and TR337/520/620modules	BMG, UK	PHERAstar ® *FSX*
ProxiPlate-384 Plus	PerkinElmer, MA, US	6008280
OptiPlate-384	PerkinElmer, MA, US	6007290
GE Amersham Typhoon^TM^	GE, US	GE Amersham Typhoon^TM^

### Resource availability

#### Lead contact

Further information and requests for resources and reagents should be directed to and will be fulfilled by the lead contact, Dmitry Veprintsev (Dmitry.Veprintsev@nottingham.ac.uk).

#### Materials availability

Plasmids generated in this study are available from the lead contact upon request, subject to the MTA with the University of Nottingham.

### Experimental model and subject details

No human subject or animal models were used in this study. The cell lines T-Rex^TM^-293 cells stably expressing pcDNA4TO-TS-SNAP-β_2_AR or pcDNA4TO-TS-SNAP- A_2A_ were used in this study. These cell lines were maintained in high glucose DMEM (Sigma D6429) with 10% foetal bovine serum (FBS), 5μg/μL blasticidin and 20μg/μL zeocin, at 37°C and 5% CO_2._

### Method details

#### Molecular biology

The construct pcDNA4TO-TwinStrep (TS)-SNAP-β_2_AR was generated by amplification of the SNAP and β_2_AR sequences of the pSNAPf-ADRB2 plasmid (NEB) and insertion into pcDNA4TO-TS using Gibson assembly (Heydenreich et al., 2017; Heydenreich et al., 2020). The construct pcDNA4TO-TS-SNAP-A_2A_ was generated by amplifying the A_2A_ receptor from the pDNA3.1 SNAP A_2A_ construct described in (Comeo et al., 2020) and inserting into pcDNA4TO-TS-SNAP vector using Gibson assembly. This therefore gave the construct pcDNA4TO-TS-SNAP-A_2A._ Both constructs used a signal peptide based on the 5HT_3A_ receptor to increase protein folding and expression.

#### Transfection and mammalian cell culture

pcDNA4TO-TS-SNAP-β_2_AR or pcDNA4TO-TS-SNAP-A_2A_ were stably transfected into T-Rex^TM^-293 cells (Invitrogen) using polyethylenimine (PEI). A mixed population stable line was selected by resistance to 5 μg/mL blasticidin and 20 μg/mL Zeocin. Stable cell lines were maintained in high glucose DMEM (Sigma D6429) with 10% foetal bovine serum (FBS), 5μg/μL blasticidin and 20μg/μL zeocin, at 37°C and 5% CO_2._ When ∼70% confluent TS-SNAP-β_2_AR expression was induced with 1μg/mL tetracycline. Cells were left to express for 50hrs before harvesting.

#### Labelling TS-SNAP-β_2_AR with terbium cryptate or SNAP-AlexaFluor488 or 647

Media was aspirated from T175 flasks and adherent cells washed twice at room temperature with Phosphate Buffered Saline (PBS). Adherent cells were labelled with 100nM SNAP-Lumi4-Tb labelling reagent in Labmed buffer (both Cisbio, UK) for 1 hr at 37°C and 5% CO_2,_ or for 30mins with SNAP-AlexaFluor 488 (NEB) in cell culture media. Cells were washed twice more with PBS and detached with 5mL non enzymatic cell dissociation solution (Sigma, UK). Cells were pelleted by centrifugation for 10 min at 1000*xg*, supernatant was removed, and cell pellets frozen at -80°C.

#### TS-SNAP-β_2_AR membrane preparation

Cell pellets were thawed on ice and resuspended in 20mL buffer B (10mM HEPES and 10mM EDTA, pH 7.4). Suspensions were homogenised using 6 x 1 sec pulses of a Polytron tissue homogeniser (Werke, Ultra-Turrax). Suspensions were centrifuged at 48,000x*g* and 4°C for 30 min, supernatant was removed and resuspended and centrifuged again as above. Resulting pellets were resuspended in buffer C (10mM HEPES and 0.1mM EDTA, pH 7.4) and frozen at -80°C.

#### Solubilisation of TS-SNAP-β_2_AR using DDM or DIBMA

Membranes were incubated with 3% DIBMA (w/v) (Anatrace, UK) in 20mM HEPES, 10% (v/v) glycerol, and 150mM NaCl, pH 8 at room temperature or 1% DDM (w/v), 20mM HEPES, 5% (v/v) glycerol, and 150mM NaCl, pH 8 at 4°C for 2-3 h. Samples were clarified by ultracentrifugation at 4°C for 1hr at 100,000*xg* for ligand binding assays and 16900*xg* at 4°C for 45min for thermostability assays.

#### Affinity purification of DDM or DIBMALP TS-SNAP-β_2_AR

Solubilised DDM-TS-SNAP-β_2_AR and DIBMALP- β_2_AR samples were purified using 20μL of 5% MagStrep “type3” XT magnetic beads suspension (IBA). Beads were prepared by removal of supernatant using magnetic rack and 2x washes in 200μL solubilisation buffers before resuspension in samples. Samples were incubated with beads for 2hrs at 80RPM on a roller in cold room. Supernatant was then removed from beads using magnetic rack and beads were washed twice with wash buffer (20mM HEPES, 10% glycerol, 150mM NaCl, pH 7.5 with 0.1% DDM for DDM sample only), before resuspension in 50μL elution buffer. Elution buffer consisted of 1part 10X buffer BXT (IBA) and 9 parts wash buffer. Elution took place for 2 hours at 80RPM on a roller in cold room. Sample were then separated from beads using magnetic rack.

#### Fluorescence size exclusion chromatography (FSEC)

30μL crude lysate samples of Alexa488-DDM or DIBMALP-β2AR were run of Yarra 1.8μm SEC-x300 2.5mL column (Phenomenex, CA, US) using shimadzu prominence HPLC system. Running buffer consisted of 20mM HEPEs, 150mM NaCl, 5% glycerol, and 0.03% DDM for DDM- β_2_AR sample only. FSEC took place at a flow rate of 0.2mL/min and 0.2mL fractions collected. Samples were excited at 488nm, and emission collected at 520nm. GE HMW calibration kit was use as the standard.

#### In gel fluorescence

15μL of each FSEC fraction was run on TruPage^TM^ 4-20% Bis-Tris 17 well gel using NuPage^TM^ LDS sample loading buffer with 5mM Dithiothreitol (DTT) and NuPage^TM^ MOPs running buffer. Gels were run for 50min at 200V. Samples were not boiled prior to gel electrophoresis. 5μL PageRuler^TM^ Prestained Protein Ladder (10-140kDa). was used as the ladder. Gels were scanned on GE Typhoon scanner using Fluorstage and Cy2 or Cy5 filter sets. PMT was set to auto and pixel size to 200μm.

#### TR-FRET ligand binding assays

TR-FRET between the donor Lumi4-Tb and the fluorescent acceptors 633/650 S-propranolol-red (CellAura, UK, supplied by Hello Bio, UK, cat no. HB7817) (F-propranolol) was measured by exciting at 337nm and quantifying emission at 665nm and 620nm using a PheraStar FSX (BMG Labtech) and HTRF 337 665/620 module (BMG Labtech). Assay buffer consisted of 20mM HEPES, 5% glycerol, 150mM NaCl, and 0.5% Bovine Serum Albumin (BSA), pH 8.0 for DDM solubilised samples 0.1% DDM was used. All binding assays used a final concentration of 1% Dimethyl sulfoxide (DMSO), assay volume of 30μL, 384 well OptiPlates (PerkinElmer, US) and 3μM cyanopindolol was used to determine non-specific binding (NSB). Receptors were added to plates last, and the plates were incubated at room temperature for 45 mins prior to reading. For competition binding assays 100nM of F-propranolol was used for membrane and DDM samples and 200nM F-propranolol for DIBMA samples.

#### ThermoFRET thermostability assays

Solubilised Lumi4-Tb labelled β2AR was incubated with 10μM BODIPY™ FL L-Cystine dye (Molecular Probes, U.S) with or without 200nM F-propranolol or 100μM cyanopindolol, for 15 mins on ice in 20mM HEPES, 150mM NaCl, 5% glycerol, 0.5% BSA, pH8. For DDM samples 0.1% DDM was used. 20μL samples were added to each well of a 96-well PCR plate and incubated for 30 min over a temperature gradient of 20-78°C across the plate using alpha cycler 2 PCR machine (PCRmax, U.K). Samples were transferred to a 384-well proxiplate (PerkinElmer, U.S). TR-FRET between BODIPY™ FL L-Cystine dye and Lumi4-Tb was read by exciting at 337nm and reading emission at 620nm and 520nm using Pherstar FSX and 337 520/620 module (BMG Labtech). F-propranolol and fluorescent XAC (F-XAC) (CellAura, UK) binding was measured using HTRF 337 665/620 module as above.

#### Data analysis

##### TR-FRET ligand binding data

Total and NSB for F-propranolol binding to the β_2_AR was fitted to one-site models in GraphPad Prism 8 according to Equations 1 and 2.

(Equation 1) $$Total\;binding=\;\frac{Bmax*X\;}{\left(K_{d}+X\right)}+\left[NS*X+background\right]$$

Equation 1

Where:

NS = slope of linear nonspecific binding
Background = Y when X is 0
Bmax = the maximum specific binding
K_d_ = the equilibrium dissociation constant
Y = specific binding
X= concentration of tracer

(Equation 2) NSB=[NS∗X+background]

Equation 2

Specific binding of F-propranolol to the β_2_AR was fitted to the one site specific binding model in GraphPad Prism 8 according to Equation 3. Final K_d_ values were taken as an average of K_d_ values from individual specific curve fits.

(Equation 3) $$Y=\frac{Bmax*X\;}{\left(K_{d}+X\right)}$$

Equation 3

Where:

Y = specific binding
K_d_ = the equilibrium dissociation constant of the labelled ligand

Equilibrium competition binding data was fitted to the One site Fit K_i_ model in GraphPad Prism 8 according to Equation 4 and 5. Final K_i_ values were taken as an average of individual curve fits.

(Equation 4) $$Y=\;\frac{\left(Top-Bottom\right)}{\left(1+10^{\left(x-LogIC_{50\;}\right)}\right)+Bottom}\;$$

Equation 4

Where:

Y = binding of tracer
IC_50_ = the concentration of competing ligand which displaces 50% of radioligand specific binding.

(Equation 5) $$K_{i}=\frac{IC_{50}}{1+\left(\frac{\left[L\right]}{K_{d}}\right)}$$

Equation 5

Where:

*K*_i_ = the inhibition constant of the unlabelled ligand
[L] = concentration of labelled ligand
K_d_ = the equilibrium dissociation constant of the labelled ligand.

#### ThermoFRET thermostability curves

All ThermoFRET thermostability data from each experiment was fitted to a Boltzmann sigmoidal curve using GraphPad Prism 8 according to Equation 6 to obtain a melting temperature (Tm) value. Final Tm values were taken as an average of Tm values from individual curve fits.

(Equation 6) $$Y=Bottom+\;\frac{\left(Top-Bottom\right)}{1+\exp\left(\frac{Tm-X}{Slope}\right)}$$

Equation 6

Where:

Y = the relative concentration of proteins in the unfolded state
X = Temperature (^o^C)
Tm = The temperature at which half the protein of interest is unfolded

### Quantification and statistical analysis

Comparison of *T*_m_, *K*_d_ or *K*_i_ values was made using a one-way Analysis Of Variance (ANOVA) test and Tukey’s post hoc multiple comparison test. Statistical comparison of *T*_m_ values obtained with F-propranolol Vs BODIPY™ FL L-Cystine dye was made using an unpaired t test. All statistical analysis was completed in GraphPad Prism 8 and p<0.05 was considered statistically significant.

## Data Availability

Any additional information required to reanalyze the data reported in this paper is available from the lead contact upon request.
