# Supplementary material for: Functional solubilization of the β2-adrenoceptor using diisobutylene maleic acid
Source: iScience. 2021 Oct 28;24(12):103362. doi: 10.1016/j.isci.2021.103362 (PMC8605084; doi:10.1016/j.isci.2021.103362)
Supplement: Document S1. Figures S1 and S2 [file mmc1.pdf]

iScience, Volume *24*

Supplemental information

Functional solubilization of the β_2-adrenoceptor using diisobutylene maleic acid

Clare.R. Harwood, David A. Sykes, Bradley L. Hoare, Franziska M. Heydenreich, Romez Uddin, David R. Poyner, Stephen J. Briddon, and D.B. Veprintsev

Data S1.

ORF amino acid sequences for Twin-Strep-SNAP-β_2AR and Twin-Strep-SNAP-A_{2A}R used in this study (relates to STAR methods/molecular biology)

Legend:

Signal peptide based on the 5HT3A receptor

Strep tag (TS)

SNAP tag

Linker

Human β_2AR

Human A_{2A}

Amino acid sequence for Twin-Strep-SNAP-β_2AR

```
MRLCIPQVLLALFLSMLTGPGEGSASDIGAPAFKSVQTGEFTAAAGSAWSHPQFEKGGGSGG
GSGGSAWSHPQFEKGSGGSEDLMDKDCEMKRTTLDSPLGKLELSGCEQGLHRIIFLGKGTSA
ADAVEVPAPAAVLGGPEPLMQATAWLNAYFHQPEAIEEFPVPALHHPVFQQESFTRQVLWKL
LKVVKFGEVISYSHLAALAGNPAATAAVKTALSGNPVPILIPCHRVVQGDLDVGGYEGGLAV
KEWLLAHEGHRLGKPGLGPAGTMGQPGNGSAFLLAPNGSHAPDHDVTQQRDEVWVVGMGIVM
SLIVLAIVFGNVLVITAIAKFERLQTVTNYFITSLACADLVMGLAVVPFGAAHILMKMWTFG
NFWCEFWTSIDVLCVTASIETLCVIAVDRYFAITSPFKYQSLLTKNKARVIILMVWIVSGLT
SFLPIQMHWYRATHQEAINCYANETCCDFFTNQAYAIASSIVSFYVPLVIMVFVYSRVFQEA
KRQLQKIDKSEGRFHVQNLSQVEQDGRTGHGLRRSSKFCLKEHKALKTLGIIMGTFTLCWLP
FFIVNIVHVIQDNLIRKEVYILLNWIGYVNSGFNPLIYCRSPDFRIAFQELLCLRRSSLKAY
GNGYSSNGNTGEQSGYHVEQEKENKLLCEDLPGTEDFVGHQGTVPSDNIDSQGRNCSTNDSL
L*
```

Amino acid sequence for Twin-Strep-SNAP-A_{2A}

```
MRLCIPQVLLALFLSMLTGPGEGSASDIGAPAFKSVQTGEFTAAAGSAWSHPQFEKGGGSGG
GSGGSAWSHPQFEKGSGGSEDLMDKDCEMKRTTLDSPLGKLELSGCEQGLHRIIFLGKGTSA
ADAVEVPAPAAVLGGPEPLMQATAWLNAYFHQPEAIEEFPVPALHHPVFQQESFTRQVLWKL
LKVVKFGEVISYSHLAALAGNPAATAAVKTALSGNPVPILIPCHRVVQGDLDVGGYEGGLAV
KEWLLAHEGHRLGKPGLGPAGTPIMGSSVYITVELAIAVLAILGNVLVCWAVWLNSNLQNVT
NYFVVSLAAADIAVGVLAIPFAITISTGFCAACHGCLFIACFVLVLTQSSIFSLLAIAIDRY
IAIRIPLRYNGLVTGTRAKGIIAICWVLSFAIGLTPMLGWNNCGQPKEGKNHSQGCGEGQVA
CLFEDVVPMNYMVYFNFFACVLVPLLLMLGVYLRIFLAARRQLKQMESQPLPGERARSTLQK
EVHAAKSLAIIVGLFALCWLPLHIINCFTFFCPDCSHAPLWLMYLAIVLSHTNSVVNPFIYA
YRIREFRQTFRKIIRSHVLRQQEPFKAAGTSARVLAAHGSDGEQVSLRLNGHPPGVWANGSA
PHPERRPNGYALGLVSGGSAQESQGNTGLPDVELLSHELKGVCPEPPGLDDPLAQDGAGVS*
```

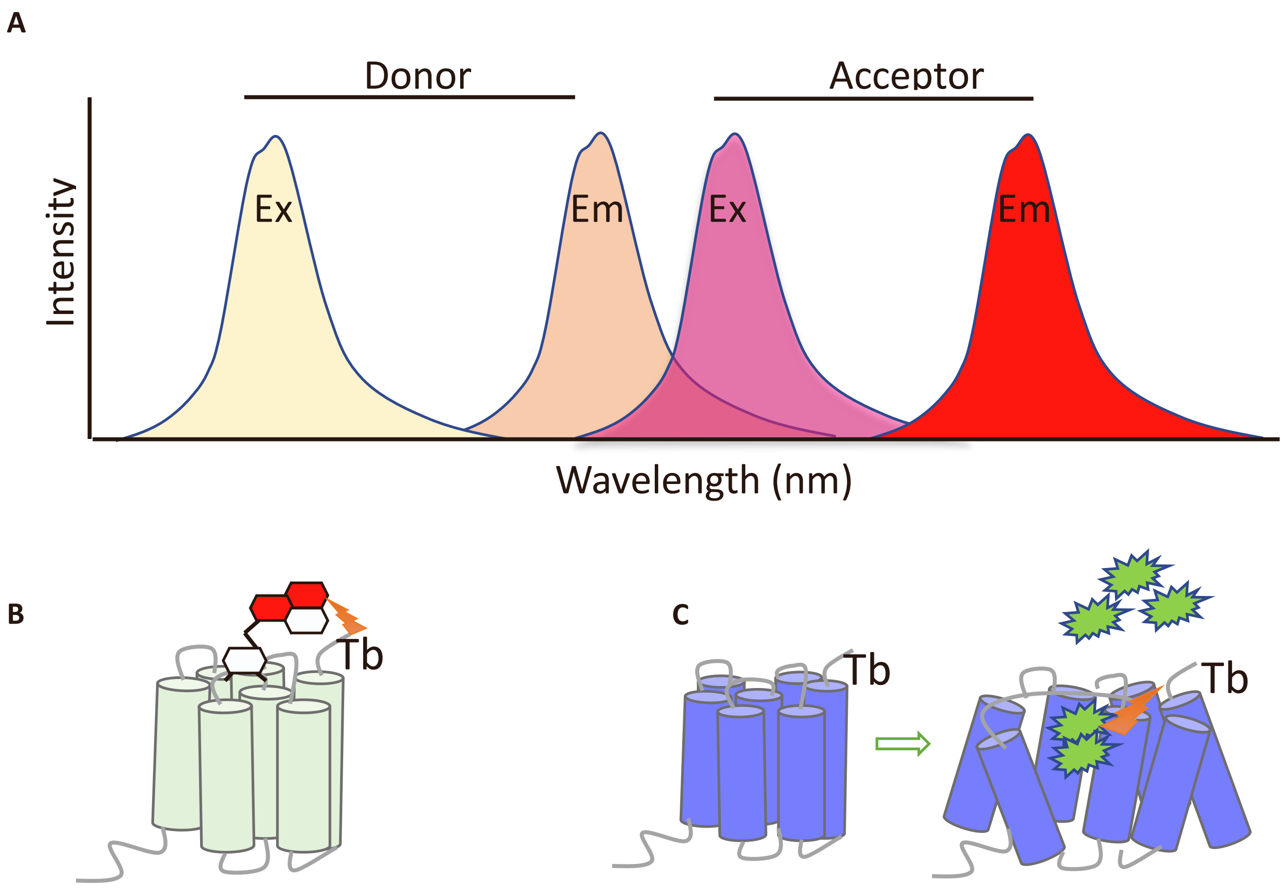

Supplementary figure 1: A summary of Förster Resonance Energy Transfer and its application in this study, **A)** FRET occurs when the emission spectra of a donor fluorophore overlaps with the excitation spectra of an acceptor fluorophore therefore exciting the acceptor fluorophore when the pair are in close proximity **B)** Time resolved FRET between Lumi4-terbium of the N terminus of the β_2AR and fluorescent propranolol was used to measure ligand binding, figures 2-3. **C)** Time resolved FRET between Lumi4-terbium on the N terminus of the β_2AR and F-L L-cysteine dye, which bound the cysteines exposed upon unfolding of the core of the β_2AR was used to measure thermostability, Figure 4.

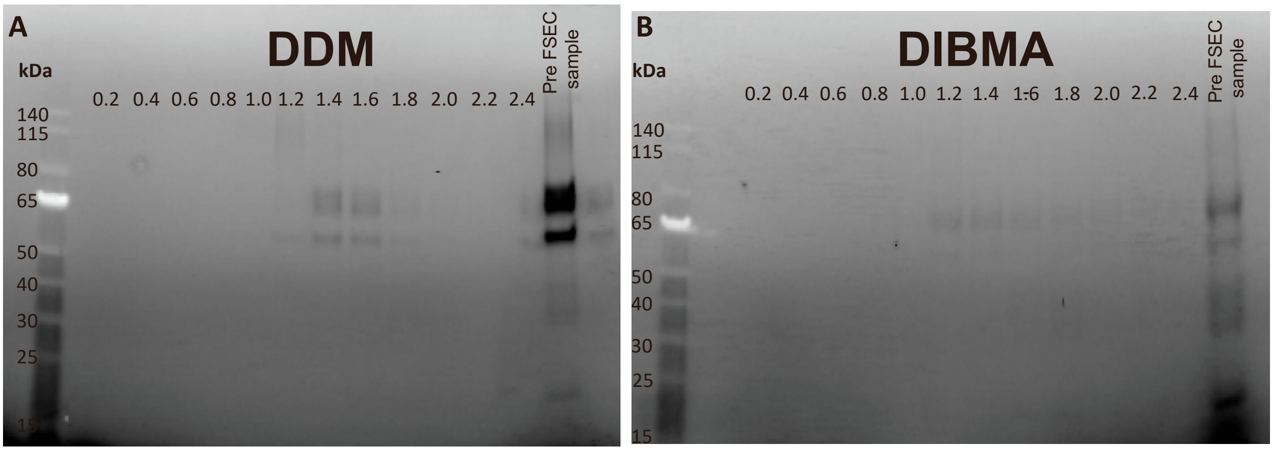

Supplementary figure 2: Confirmation of FSEC fractions (figure 1C) by in gel fluorescence (cy2). Representative example of n=3.
